# Ethanol and Volatile Fatty Acid Production from Lignocellulose by *Clostridium cellulolyticum*

**DOI:** 10.5402/2013/137835

**Published:** 2012-08-05

**Authors:** K. Williams, Y. Zheng, J. McGarvey, Z. Fan, R. Zhang

**Affiliations:** ^1^Department of Biological and Agricultural Engineering, University of California, Davis, Davis, CA 95616, USA; ^2^Novozymes A/S, Krogshoejvej 36, 2880 Bagsvaerd, Denmark; ^3^Plant Mycotoxin Research Unit, ARS, USDA, Albany, CA 94710, USA

## Abstract

*Clostridium cellulolyticum* is capable of producing glycosyl hydrolase enzymes as well as fermentation products including ethanol and acetate. In this study, the potential of using *C. cellulolyticum* for ethanol and volatile fatty acid production from straw and grape pomace was examined. For rice straw, the effects of alkaline pretreatment and substrate sterilization prior to fermentation on products yields were also investigated. Effects of alkaline pretreatment and necessity for subsequent washing were tested for two types of grape pomace. For rice straw, the highest ethanol yield was 0.16 g/gVS from the straw pretreated with 10% sodium hydroxide loading at 121°C for 1 hour. Sterilization of the straw prior to fermentation was found to be not significant for ethanol production. Sterilization appeared to decrease native acetogen populations in the rice straw, resulting in lower acetic acid yields. The highest ethanol yield from grape pomace was of 0.09 g/gVS from the pretreated pomace. Pomace type (red or white) and washing were found to be not significant. Ethanol yields by *C. cellulolyticum* were lower than those from yeast in a simultaneous saccharification and fermentation system, but overall conversion of cellulose and hemicellulose was high, between 68 and 79%.

## 1. Introduction

Lignocellulosic biomass, including crop and forestry residues, grasses, and other plant materials, is currently the primary focus feedstock for bioethanol and biochemical production research. This study focused on two lignocellulosic substrates that are common in California: rice straw and grape pomace. In California, there are around 200,000 hectares of rice planted resulting in around 1.1 million tons of rice straw per year [1]. The rice straw is usually burned or tilled into the soil. Tilling can be cost intensive, and burning causes significant air pollution, which is highly regulated in California. An environmentally friendly and economic use for this agricultural residue is needed. The rice straw used in this study was composed of 16.8% lignin, 38.8% cellulose, 24.1% hemicellulose, and 20.4% ash on a dry basis with a moisture content of 5.8%. The hemicellulose fraction was made up of 83.3% xylose, 1.3% galactose, and 15.4% arabinose. Grape pomace is also abundant in California with around 150,000 dry tons of grape pomace produced each year in California [2]. There are two types of grape pomace: white (produced from grape crushing prior to fermentation) and red (resulted after fermentation). The white pomace used in this study contained 16% lignin, 8.9% cellulose, 5.8% hemicellulose, and 10% ash on a dry basis with moisture content of 64%. The red pomace was composed of 16% lignin, 10.5% cellulose, 8% hemicellulose, and 15% ash on a dry basis with a moisture content of 59% [3].

Due to the presence of cellulose, hemicellulose, and lignin, the complex nature of these materials makes lignocellulosic ethanol processing considerably more complicated than corn ethanol. Pretreatment, enzyme production, hydrolysis, and fermentation are the main four steps of the currently uneconomical lignocellulosic ethanol production process [4, 5]. Three ways to potentially increase the economical efficiency of lignocellulosic ethanol processing are to (i) decrease the number of steps [6–9], (ii) utilize all portions of the material [10], and (iii) produce high value coproducts [10, 11].

Combining the enzyme production, hydrolysis, and fermentation steps into a single step, called consolidated bioprocessing (CBP), has potential to contribute towards the first idea in the above list. Many *Clostridium* ssp. have promise for use in a CBP system due their ability to produce glycosyl hydrolase enzymes and ferment the released sugars to ethanol and other useful products. *C. cellulolyticum* produces mostly ethanol and acetic acid [12–15]. Acetic acid can be used to make chemicals like vinyl acetate monomer (VAM), acetic anhydride, and terephthalic acid [16]. Previous studies show ethanol and acetic acid yields of 0.14 and 0.19 g/g from xylan [17]. *C. cellulolyticum* can potentially utilize all of the carbohydrate portions of the biomass, but the lignin portion is unfermentable. Lignin can be burned for energy or turned into coproducts such as resins and glue [18] but needs to be separated from the carbohydrate portions of the biomass. Alkali pretreatment has been shown to effectively remove lignin but leave the carbohydrate portions relatively intact [19, 20]. The positive effects of alkali pretreatment of rice straw and grape pomace have been demonstrated [21–23], but little work with CBP or *Clostridium*-based systems has been conducted. This study will compare ethanol yields from sodium hydroxide pretreated rice straw and grape pomace using *C. cellulolyticum* in a CBP system.

Removing the substrate sterilization step is another way to streamline the production process. The purpose of substrate sterilization is to kill native microbes present on the substrate, which can lower ethanol production. Native microflora competes with the process microbe, *C. celluloyticum* in this case, and lower final yields of desired product [24]. Alternative sterilization methods, such as hydrogen peroxide [24], or process development that limits negative effects of contamination are needed. This study investigated the effects of eliminating the substrate sterilization step entirely in rice straw ethanol production.

Post-pretreatment washing is another potential redundant step in the ethanol production process. Fermentation pH has been shown to effect the ethanol production by mixed anaerobic cultures [25] and by *Clostridium thermosaccharolyticum* [26]. In both cases, neutral pH leads to higher ethanol production. After sodium hydroxide pretreatment, the substrate pH is much higher than neutral and could potentially affect the fermentation yields. Washing the pretreated solids can help neutralize the pH but also adds another step to the total process. This research investigated the effects of eliminating the washing step after alkali pretreatment of grape pomace.

Overall, the objectives of this research were to address the three factors listed above through characterization of the product yields of *C. cellulolyticum* growing on rice straw and grape pomace. *C. cellulolyticum* can be used in a CBP system that combines several processing steps, with potential to ferment all carbohydrate portions of the substrate and produces valuable coproducts. Along the way, experiments also revealed the effects of alkali pretreatment, substrate sterilization, and post-pretreatment washing on the product yields.

## 2. Materials and Methods

### 2.1. Pretreatment

 The rice straw was hammer-milled to 1 cm particle size. The hammer-milled straw (20 grams dry weight) was mixed with 200 mL of 10% (w/v) sodium hydroxide. The mixture was then either autoclaved at 121°C for two hours (high-temperature pretreatment) or incubated at 25°C for 24 hours (low-temperature pretreatment). These pretreatments were chosen as a result of optimization experiments conducted in our lab. After pretreatment, the solids were separated from the liquid and then washed with deionized water until the pH reached 7.0. Finally, the rice straw was dried at 45°C for 48 hours so that it could be ground to 20 mesh (841 *μ*m) and stored at room temperature without spoiling. A raw straw control was also investigated using hammer-milled straw that was ground to 20 mesh.

The grape pomace (20 grams dry weight) was pretreated with 200 mL of 1% (w/v) sodium hydroxide for 24 hours at 25°C and ambient pressure. Two different post-pretreatment methods were explored for the grape pomace. Grape pomace contains residual free sugars that can be lost during the washing step, but it is also important to adjust the pH to close to neutral for fermentation by the *Clostridium* bacteria. The first washing method was rinsing with deionized water until the pH was back to 7.0. The second method was pressing the excess pretreatment liquid from the solids and using them as is.

### 2.2. Media and Bacteria Strains

Stock cultures of 250 mL were grown up on cellobiose to an optical density (OD) of 0.7–0.9. The experimental tubes were then inoculated to a starting OD of around 0.1 in a 10 mL working volume. The media composition for both stocks and experiments was as follows (mg/L): 343.5 K_2_HPO_4_, 450 KH_2_PO_4_, 367.5 NH_4_Cl, 900 NaCl, 157.5 MgCl_2_·6H_2_O, 120 CaCl_2_·2H_2_O, 0.75 MnCl_2_·4H_2_O, 0.75 CoCl_2_·4H_2_O, 5.2 Na_2_EDTA, 1.5 FeCl_2_·4H_2_O, 0.07 ZnCl_2_, 0.1 MnCl_2_·4H_2_O, 0.062 H_3_BO_4_, 0.192 CoCl_2_·6H_2_O, 0.017 CuCl_2_·6H_2_O, 0.024 NiCl_2_·6H_2_O, 0.036 Na_2_MoO_4_·2H_2_O, 0.001 Rezazurin, 2,000 yeast extract, 1,000 cysteine HCl (reducing agent), 20,000 NaHCO_3_. The media was boiled to remove dissolved oxygen and purged with carbon dioxide before being sealed in 500 mL round bottom flasks (stocks) or 20 mL Hungate tubes (experiments). Stocks were then autoclaved, while experimental tubes were autoclaved according to the experimental designs described as follow. The bacteria strain used in these experiment was *Clostridium cellulolyticum* (ATCC number 35319). Cultures were incubated at 37°C, and the culture volume was 10 mL with an initial substrate loading of 4 gVS/L. Samples were taken at 5, 10, and 20 days. The 20-day data are presented for all substrates.

### 2.3. Rice Straw Experimental Design and Statistical Analysis

This set of experiments had two objectives: first, to investigate the ability of *C. cellulolyticum* to grow on rice straw. Usually, the substrate is autoclaved with the media before inoculation of the *Clostridium* bacteria. However, this introduces what could be a “second pretreatment” effect and may not give a true estimate of ethanol potential of original pretreated rice straw. Therefore, the second objective was to investigate the effects and necessity of autoclave sterilization on the product yields.

There were two factors for the experimental design: rice straw pretreatment and autoclave sterilization (121°C) for 30 minutes, as shown in Table 1. Treatments 1–3 sterilized the media in the autoclave first and then the rice straw added afterwards, while 4-5 autoclaved the rice straw with the media. Treatments 1 and 2 allowed insight into how the high- and low-temperature pretreated straws compare when not autoclave sterilized with the media, while treatments 4 and 5 allowed comparison of high temperature pretreated and raw rice straw when both are sterilized with the media. A comparison of the double autoclaving with just the pretreatment autoclaving can be made with treatments 1 and 5. A control with *C. cellulolyticum* and no straw was subtracted from each treatment. All treatments and controls were run in triplicate.

Statistical analysis on ethanol and acetic acid yields was conducted using ANOVA and Tukey tests with R software (CRAN). Both models contained both factors and the interaction giving the following:

(1) Ethanol=RiceStraw+Sterilization+  RiceStraw∗Sterilization,

(2) AceticAcid=RiceStraw+Sterilization+RiceStraw∗Sterilization.

### 2.4. Grape Pomace Experimental Design and Statistical Analysis

The main objective of these experiments was to assess the product yields by *C. cellulolyticum* growing on grape pomace. Two types of grape pomace, red and white, were used. Each type of pomace was either left raw or pretreated with sodium hydroxide. The pretreated grape pomace was either washed or pressed as described in the pretreatment section. The experimental design for the experiment, shown in Table 2, allowed for comparisons of two different types of pomace (red versus white), raw versus pretreated pomace, and washed versus pressed pomace after the pretreatment. All treatments were sterilized for 30 minutes in the autoclave (121°C) prior to inoculation with *C. cellulolyticum* and run in triplicate.

Statistical analysis on the ethanol and acetic acid yields was completed in R using the net day-20 data. The factors were sorted as shown in Table 2. The analysis began with the full model, including substrate, pretreatment, and all interaction terms. For the ethanol yields, substrate was just short of significant, but the interaction between substrate and pretreatment was significant so both were left in the model to give

(3) Ethanol=Substrate+Pretreatment+Substrate∗Pretreatment.

ANOVA tests on the acetic acid yields showed that the interaction term was not significant, leaving

(4) $$\begin{array}{c} \text{AceticAcid}=\text{Substrate}+\text{Pretreatment}. \end{array}$$

### 2.5. Product Analysis

Product yields including ethanol, lactic acid, acetic acid, formic acid, propionic acid, butyric acid, and valeric acid were measured using high-performance liquid chromatography (HPLC). The analysis was run on a Shimadzu HPLC system with Biorad HPX-87H ion exclusion column, Microguard cation H guard column, and 0.6 mL/min flow rate of 15 mmol sulfuric acid with an 85°C separation temperature. A refractive index detector was used for ethanol, and sugars while a photo diode array detector at 205 nm was used for volatile fatty acids.

## 3. Results

### 3.1. Rice Straw

**Figure 1:**
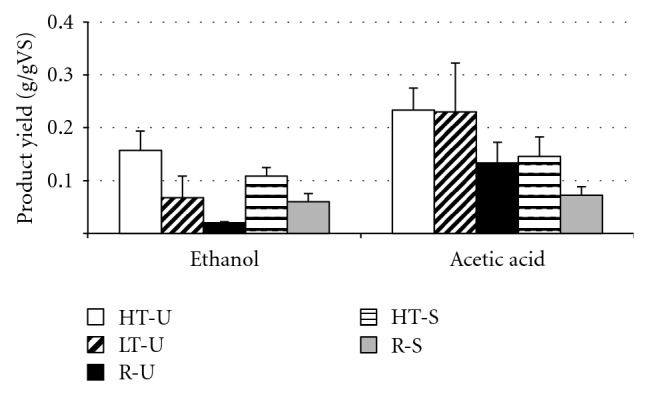
*C. cellulolyticum* product yields after 20 days from rice straw that was high-temperature pretreated, unsterilized (HT-U: white), low-temperature pretreated, unsterilized (LT-U: diagonal lines), raw, unsterilized (R-U: black), high-temperature pretreated, sterilized (HT-S: horizontal lines), high-temperature pretreated, sterilized (R-S: grey).

The product yields for *C. cellulolyticum* growing on rice straw are shown in Figure 1. The control was subtracted out from each treatment. Ethanol and acetic acid were the main two products. The highest ethanol yield was 0.16 g/gVS, also from the unsterilized high temperature pretreated rice straw. Other products were isobutyric and butyric acid, with yields of 0.05 g/gVS each, from unsterilized high-temperature pretreated rice straw. The highest total product yield was 0.47 g/gVS for the unsterilized high-temperature pretreated straw, which represents 68% conversion of the cellulose and hemicellulose. Ethanol yields got progressively higher as the pretreatment becomes more severe for the nonsterilized rice straw. For the sterilized straws, the difference in ethanol yield between the high-temperature pretreated and raw straws was smaller, suggesting that there is an interaction between sterilization and pretreatment.

The ANOVA tests showed that the rice straw term (from (1)) was the most significant term in the model (*P* = 0.0074). The sterilization factor was highly insignificant (*P* ≫ 0.05), but the interaction term was almost significant with a *P* value of 0.086. The low-interaction *P* value combined with the above data trends seemed to warrant further investigation of the RiceStraw∗Sterilization interaction using Tukey comparisons so both terms were left in the model. Tukey tests on the temperature factor revealed that the high-temperature pretreated rice straw treatment was significantly (*P* ≤ 0.05) different from both the low-temperature pretreated and raw rice straw. However, the low-temperature and raw rice straws were not significantly different (*P* ≤ 0.05). This suggests that high-temperature treatment is necessary to increase the *C. cellulolyticum* ethanol yield to a value significantly (*P* ≤ 0.05) higher than the raw rice straw. Tukey tests for the sterilization factor showed no significant difference between sterilized and unsterilized straws.

Tukey comparisons for the pretreatment-sterilization interaction term are shown in Table 3. The low-temperature pretreatment was not different from any other treatments. The high-temperature pretreated sterilized straw was not significantly different from the raw sterilized straw, but it was significantly higher than the raw unsterilized straw. The sterilized raw straw ethanol yield is significantly lower than that of the unsterilized high-temperature pretreated straw, but not the sterilized high-temperature pretreated straw.

The highest acetic acid yield was 0.23 g/gVS from the unsterilized pretreated rice straw (high- and low-temperature pretreated yields were similar). Acetic acid yield for the sterilized high-temperature pretreated straw was around 0.13 g/gVS. ANOVA tests revealed that rice straw and sterilization were both significant in the model. This contrasts with the ethanol yields for which sterilization was not significant.

 Tukey tests over sterilization showed that the sterilized straw acetic acid yields were significantly lower than those of nonsterilized straw. Tukey tests over rice straw type showed that raw straw acetic acid yields are significantly lower than those from low-temperature pretreated straw, but not the high-temperature pretreated straw. However, this could be misleading since there is not a sterile low-temperature rice straw. For this reason, the interaction term was left in the model so that specific treatments could be compared, even though it was not statistically significant. Tukey comparisons for the interaction term are shown in Table 4. The “A” Tukey group contains treatments from all rice straw levels. The raw sterilized straw is significantly lower than the low- and high-temperature pretreated unsterilized straw, but not the high temperature sterilized straw or the raw unsterilized straw.

### 3.2. Grape Pomace

**Figure 2:**
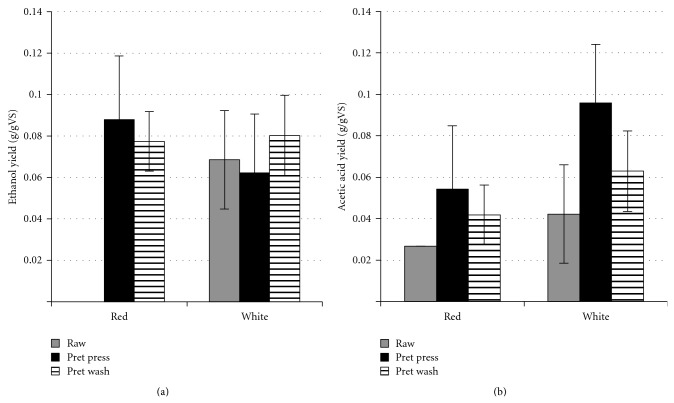
Net ethanol (a) and acetic acid (b) yields by *C. cellulolyticum* after 20-day fermentation on red and white grape pomace that was raw (raw: grey), pretreated and pressed (pret press: black), and pretreated and washed (pret wash: horizontal lines).

The main products of *C. cellulolyticum* on grape pomace were ethanol and acetic acid. Small amounts of propionic, butyric, and valeric acids were also produced. The raw red grape pomace appeared to have the highest ethanol yield of 0.13 g/gVS, but it also had the highest initial ethanol concentration. The net ethanol production was actually slightly negative over time. All other treatments resulted in a net increase in ethanol yield over time. In order to compare the net increase in ethanol produced by *C. cellulolyticum*, the initial ethanol was subtracted from the 20-day data, with the negatives set to zero. The net ethanol production data are shown in Figure 2. All of the ethanol yields are similar except for the raw red pomace, which had no net increase in ethanol production. The highest ethanol yield of 0.09 g/gVS was from the pretreated and pressed red pomace. The pretreated and pressed white pomace had the highest total product yield of 0.16 g/gVS.

 Tukey analysis on the pretreatment factor revealed that pretreated grape pomace resulted in significantly (*P* ≤ 0.05) higher ethanol yield by *C. cellulolyticum* than raw pomace. The ethanol yield from the washed pomace was not significantly different (*P* ≤ 0.05) from the pressed. The type of pomace (red or white) also did not significantly (*P* ≤ 0.05) change the ethanol yield. Tukey analysis on the interaction term revealed that the raw red pomace was significantly lower than the raw white, pretreated red, and pretreated white pomace. No other significant differences existed within the interaction.

 The highest net acetic acid yield from grape pomace was 0.10 g/gVS from the pretreated and pressed white pomace. Both types of pomace followed the same trend with pretreated pressed being the highest, followed by the pretreated washed and the raw being the lowest. Tukey tests on the acetic acid yield data showed that white pomace acetic acid yields were significantly higher (*P* ≤ 0.05) than those of the red pomace. For the pretreatment factor, the raw pomace was significantly less than the pretreated pressed pomace but not the pretreated washed.

## 4. Discussion

Ethanol and acetic acid were the main two products from *C. cellulolyticum* growing on rice straw, which was expected based on existing literature [12, 17, 27]. In previous studies, *C. cellulolyticum* had an ethanol yield of 0.15 g/g from xylan [28]. In light of this, the rice straw ethanol yield seems reasonable. Ethanol yields increased with increasing pretreatment severity for the unsterilized rice straw but for the sterilized straws, the difference between the high temperature pretreated and raw straws appears to be less significant. For raw straw, sterilization increased the ethanol yield to a level comparable to the ethanol yield of high-temperature pretreated sterilized straw. The high-temperature pretreatment in addition to autoclave sterilization did not add any benefit to the ethanol yield above sterilized raw straw. These three facts suggest that the autoclave sterilization is acting as a pretreatment to increase ethanol yields. Autoclaving of municipal waste at 130°C has been shown to solubilize hemicellulose and cellulose [29] so it seems reasonable that autoclaving straw at 121°C would release soluble carbohydrates in a similar manner. Conversely, sterilization appears to kill native acetogens leading to lower acetic acid yields. Rice straw residues have been shown to have high numbers of bacteria from the Firmicutes phyla [30], which includes acetic acid producing *Clostridium* species. The high acetic acid yields from the nonsterilized rice straws suggest production by these native rice straw microbes in addition to the *C. cellulolyticum*.

Literature shows that solid-state fermentation by *S. cerevisiae* PM-16 of red pomace at 28°C for 48 hours produced 0.05 g ethanol/g wet weight [31]. The final yield is comparable to the current study, but the fermentation time is much shorter. The net ethanol yield from raw red pomace was significantly lower than all other treatments but the raw red grape pomace also had the highest initial ethanol concentration (0.70 g/L). The high initial concentration may have been inhibitory to the growth and activity of the *C. cellulolyticum*. This suggests that the improvement from the pretreatment may be due to decreased ethanol inhibition and not an actual pretreatment effect. The lack of improvement in ethanol yield between raw and pretreated white pomace also supports this hypothesis, especially since the structural compositions of the two pomaces are similar. For acetic acid production, the pretreatment factor was significant and yields were increased with the addition of a pretreatment step for both types of pomace. The high initial ethanol concentration in the raw red pomace does not seem to affect the acetic acid production by *C. cellulolyticum*.

Pretreated rice straw had the highest ethanol production of the three substrates with a final yield of 0.16 g/gVS, which was almost twice the amount of the grape pomaces. This is most likely due to the higher cellulose and hemicellulose content in the rice straw. An ethanol yield of 0.16 g/gVS from pretreated rice straw represents 40% of the maximum theoretical yield based on the cellulose and xylan content of the straw. Ethanol production by *Saccharomyces cerevisiae* at 5.75 and 30°C was 19.2 g/L from alkali pretreated rice straw hydrolysate containing 50 g/L sugars, for a yield of 0.384 g ethanol/g sugar [32]. Reference [33] produced 25.56 g/L ethanol from alkali pretreated rice straw containing 60 g/L reducing sugars, for a yield of 0.43. Reference [23] reported 52.8% maximum theoretical yield from sodium hydroxide pretreated rice straw (8 g NaOH/100 g straw, dry weight basis) in a 96-hour simultaneous saccharification and fermentation (SSF) process using enzymes and *Saccharomyces cerevisiae*.

Ethanol yields from *C. cellulolyticum* are lower than the current SSF technologies, but the total product yields in this study show that rice straw and grape pomace have potential for combined ethanol and volatile fatty acid production. The total product yield for rice straw was 0.47 g/gVS or 68% conversion of the cellulose and xylan fractions. For grape pomace, the total product yield was 0.16 g/gVS or 79% conversion of cellulose and xylan fractions. *C. cellulolyticum* may have applications in a volatile fatty acid biofuels platform as suggested by Zhu et al. [23], in which volatile acids from microbial fermentation are hydrogenated chemically into ethanol and other alcohols. Hydrogen could be derived from thermochemical treatment of the leftover lignin fraction of the biomass [34]. Further economic analysis should be conducted to determine if acetic acid is more valuable as a chemical coproduct or as a substrate for chemical conversion to ethanol.

 Feedstock sterilization and pretreatment are two potentially energy intensive steps for lignocellulosic biofuel production. Autoclave sterilization was found to increase the ethanol yields for raw rice straw, but not for high-temperature pretreated rice straw. At industrial scale, the ability to use unsterilized rice straw would remove a high cost step and increase process efficiency. High-temperature pretreatment also has high energy input. Ethanol production from unsterilized low-temperature pretreated straw was not significantly different from the high-temperature pretreatment or the raw straw. This fact, combined with the need for low energy processing, suggests that low-temperature pretreatment without sterilization may be the best straw preparation.

## 5. Conclusion

 The rice straw pretreated with 10% sodium hydroxide (dry basis loading relative to straw) at high-temperature (121°C) for one hour had the highest ethanol (0.16 g/gVS) and acetic acid (0.23 g/gVS) yields of all the treatments tested. However, a low-temperature pretreatment with 10% sodium hydroxide at 25°C for 24 hours may be more economical if energy costs are taken into consideration. Further process design and economic analysis are needed to verify this hypothesis. Sterilization of raw straw by 30 minutes of autoclaving at 121°C acted as a pretreatment, increasing ethanol production to that of a low-temperature sodium hydroxide pretreatment. Of the grape pomace treatments, red pretreated pomace had the highest ethanol yield of 0.09 g/gVS while white pretreated pomace had the highest acetic acid yield of 0.10 g/gVS. Pressing to removing the chemical solution from the pretreated pomace is preferable over washing the pretreated pomace with water since it has lower energy and water use and there were no statistical differences between the product yields. 

## Figures and Tables

**Table 1:** Experimental design for *C. cellulolyticum* on rice straw.

Treatment	Ricestraw	Straw sterilization
1	High temperature	Unsterilized
2	Low temperature	Unsterilized
3	Raw	Unsterilized
4	Raw	Sterilized
5	High temperature	Sterilized

**Table 2:** Experimental design for *C. cellulolyticum* on grape pomace.

Treatment	Substrate	Pretreatment
1	White GP	Raw
2	Red GP	Raw
3	White GP	Pretreated washed
4	Red GP	Pretreated washed
5	White GP	Pretreated pressed
6	Red GP	Pretreated pressed

**Table 3:** Tukey comparisons for *C. cellulolyticum* ethanol yields from rice straw.

Treatment	Ethanol yield (g/g)	Tukey groups^a^
HT: U	0.157	A
HT: S	0.108	AB
LT-U	0.067	ABC
Raw: S	0.060	BC
Raw: U	0.020	C

^a^Treatments with the same letter are not significantly different.

**Table 4:** Tukey comparisons for* C. cellulolyticum* acetic acid yields from rice straw.

Treatment	Acetic acid yield (g/g)	Tukey groups^a^
HT: U	0.233	A
LT-U	0.230	A
HT: S	0.136	AB
Raw: U	0.133	AB
Raw: S	0.072	B

^a^Treatments with the same letter are not significantly different.
